# Functional Requirements of Niacinamide for Intestinal Health and Growth Performance of Nursery Pigs

**DOI:** 10.3390/ani15233415

**Published:** 2025-11-26

**Authors:** Qinyu Tan, Yunlong Shi, Dong Xu, Jiali Wang, Ziyi Yang, Sung Woo Kim, Xi Lin, Pengfei Gao, Chunbo Cai, Xiaohong Guo, Guoqing Cao, Bugao Li, Yan Zhao

**Affiliations:** 1 College of Animal Science, Shanxi Agricultural University, Jinzhong 030801, China; tan13550876667@outlook.com (Q.T.); shiyunlong1218@163.com (Y.S.); 18435749890@163.com (D.X.); 17835345580@163.com (J.W.); 18636911516@163.com (Z.Y.); gpf800411@126.com (P.G.); caichunbo@sxau.edu.cn (C.C.); g_xiaohong@126.com (X.G.); anniecao710502@aliyun.com (G.C.); 2 Department of Animal Science, North Carolina State University, Raleigh, NC 27695, USA; sungwoo_kim@ncsu.edu (S.W.K.); xilin@ncsu.edu (X.L.)

**Keywords:** low-protein diet, metabolomic, microbiota, pigs, ZnO

## Abstract

Low-protein diets are increasingly used in nursery pigs to reduce nitrogen excretion and control post-weaning diarrhea. Niacinamide, as a bioavailable form of niacin, improves protein metabolic regulation and nitrogen utilization via promoting energy metabolism, redox balance, and intestinal integrity. In addition, synergistic effects in enhancing anti-inflammation and protecting against environmental stress are reported by combining niacinamide and Zn. The levels of dietary niacinamide for optimal growth and intestinal health were determined in two experiments using nursery pigs fed a low-protein diet with and without the addition of 0.2% ZnO. The diets were supplemented with 0, 30, 130, 230, or 330 mg/kg niacinamide. In Exp. 1, niacinamide quadratically improved (*p* < 0.05) growth performance with optimal effects at 50 mg/kg, increased hemoglobin (HGB) and mean corpuscular volume, and decreased diarrhea occurrence (*p* < 0.05) at 140 mg/kg in the diet with ZnO. In Exp. 2, niacinamide improved feed efficiency (*p* < 0.05) peaked at 130 mg/kg, and serum HGB and immunoglobulin A (*p* < 0.05) peaked at 170 mg/kg while decreasing diarrhea occurrence (*p* < 0.05) by up to 315 mg/kg in the pigs with no ZnO. The level of niacinamide required for optimal growth and intestinal health was associated with Zn availability.

## Simple Summary

The optimal level of supplemental niacinamide for a low-protein diet (17.5%) was examined in nursery pigs. The effect of Zn availability on the level of niacinamide supplementation was also evaluated by testing the effects of adding 0.2% ZnO to the diet. The pigs were fed the diets individually or in groups supplemented with either 0, 30, 130, 230, or 330 mg/kg niacinamide. The growth performance, blood parameters, intestinal morphology, colonic microbiota, short-chain fatty acids, and metabolomic profiles, as well as diarrhea, were evaluated after feeding the diets for 14 or 28 days. The levels of niacinamide supplementation for optimal growth performance, intestinal health, and functional metabolic activity were estimated based on multiple evaluations. The estimated optimal levels of niacinamide were lower in diets with ZnO addition.

## 1. Introduction

Weaning is a critical period in the early life of pigs, typically occurring around 21 days of age when the intestinal tract, immune system, and intestinal microbiota are still immature [1,2,3]. The abrupt transition from maternal milk to solid feed, along with environmental and social stressors, often leads to weaning stress syndrome, which is characterized by intestinal barrier dysfunction, microbial imbalance, diarrhea, and reduced growth performance [4,5].

Pharmacological doses of ZnO have traditionally been used to alleviate post-weaning diarrhea due to their antimicrobial and anti-inflammatory effects [6,7,8]. However, environmental concerns about zinc accumulation in soil and water have led to restrictions on its use in many countries [9,10]. This shift underscores the need for effective and sustainable nutritional strategies to support gut health during the post-weaning period. Niacinamide, the amide form of niacin, plays an essential role in energy metabolism and cell repair [11,12]. Recent evidence suggests that niacinamide can enhance intestinal morphology, improve epithelial barrier integrity, and promote microbial balance [13,14,15], making it a potential nutritional candidate to mitigate weaning-associated intestinal dysfunction.

Concurrently, low-protein diets have been increasingly adopted to reduce nitrogen excretion and control post-weaning diarrhea by limiting undigested protein fermentation in the hindgut of nursery pigs [16,17,18]. However, lowering dietary protein often reduces soybean meal inclusion. Soybean meal serves as the main source of available niacin, with approximately 34 mg/kg of free niacin [19,20]. Cereal grains such as maize, commonly used in such diets, contain niacin mainly in a bound, poorly available form [21,22]. Negative impacts of a low-protein diet on Zn absorption and retention were also observed [23,24]. As a result, nursery pigs on low-protein diets may face an increased risk of suboptimal niacin intake and zinc deficiency.

Previous studies have shown that niacin or niacinamide supplementation can improve intestinal morphology, antioxidant capacity, microbial composition, and immune function in nursery pigs [14,25,26]. However, most of these studies were conducted under standard protein conditions and used limited dosage levels. Thus, the optimal dietary level under more challenging nutritional conditions is not clear. There is a lack of comprehensive dose–response evaluations of niacinamide in low-protein diets, especially under different Zn levels.

Therefore, we hypothesized that the current niacin requirement may be insufficient for nursery pigs fed low-protein diets with no Zn supplementation. Increasing niacinamide level may help compensate for reduced niacin availability and the potential Zn deficiency, further enhancing intestinal health and unleashing growth potential. To test our hypothesis, two experiments were conducted to determine the functional requirements and optimal levels of dietary niacinamide in nursery pigs fed low-protein diets with or without ZnO supplementation. The effects of increasing niacinamide supplementation on intestinal morphology and microbial composition, as well as growth performance, were evaluated. The potential optimized niacinamide level based on these evaluations is suggested for the practical feeding systems.

## 2. Materials and Methods

### 2.1. Animals and Diets

All animal procedures were performed in accordance with the ethical guidelines of Shanxi Agricultural University. Experimental authorization was obtained from the university’s Institutional Animal Welfare Committee (Approval ID: SXAU-EAW-2022P.ZO.004004186). The niacinamide supplement used in the trial was sourced from Shanghai Meinong Biotechnology Corporation (Shanghai, China).

Two trials were carried out at the Swine Research Farm of Shanxi Agricultural University (Jinzhong, China). In the first experiment, a total of 30 nursery pigs (Duroc × Landrace × Yorkshire) were chosen at 21 days of age, with an average body weight (BW) of 6.1 ± 0.1 kg. The pigs were randomly divided into 5 dietary treatments, 6 pigs (3 barrows and 3 gilts)/treatment. All pigs were housed individually and received diets containing 1600 mg Zn in the form of ZnO (Table 1) supplemented with different levels of niacinamide: 0NAM (control diet without niacinamide supplementation), 30NAM (30 mg/kg of niacinamide), 130NAM (130 mg/kg of niacinamide), 230NAM (230 mg/kg of niacinamide), and 330NAM (330 mg/kg of niacinamide). The addition level of Zn followed the official Chinese recommended guideline for weaning pigs in the first two weeks post-weaning period [27] and referred to the results summarized in part 6 of NRC (2012) nutrient requirement for trace minerals [28]. The niacinamide supplementation dosages were also determined with reference to the NRC (2012) guideline (30 mg/kg) as well as results from prior research [13,14,28,29]. All other nutrients followed the NRC (2012) swine nutritional requirement [28], with the detailed dietary ingredient composition and nutrient concentrations provided in Table 1. The vitamin premixes were produced by a commercial supplier, with niacinamide incorporated into the premix to achieve final dietary concentrations of 0, 30, 130, 230, and 330 mg/kg. Representative samples were collected from each batch after mixing, and the nutrient composition of the diets was determined using the analytical procedures outlined in the AOAC (2005) guidelines [30]. The experiment lasted for 14 days, and the environmental conditions, including room temperature, were maintained consistently at approximately 28 °C during the experiment.

In Exp. 2, a total of 64 nursery pigs (Duroc × Landrace × Yorkshire, 32 barrows and 32 gilts), with an initial average BW of 7.9 ± 0.7 kg at 28 days of age, were selected and randomly allocated to 4 dietary treatments. All pigs were group-housed—4 pigs/pen and 4 pens/treatment. Within each pen, half of the pigs were barrows and half were gilts, ensuring an approximately 1:1 sex ratio across all treatments. The pigs were fed diets containing no addition of ZnO supplemented with different levels of niacinamide: 30N (30 mg/kg niacinamide as the control group), 130N (130 mg/kg niacinamide), 230N (230 mg/kg niacinamide), and 330N (330 mg/kg niacinamide). The supplementation levels were based on the NRC (2012) recommendation (30 mg/kg) and findings from previous studies [13,14,28,29] as well as results from Exp. 1. The experimental diets were formulated following the NRC (2012) standard for nursery pigs [28], and the composition and nutritional levels are presented in Table 2. Vitamin premixes containing the specified levels of niacinamide were prepared by a certified commercial feed manufacturer. For quality assurance, representative feed samples from each batch were collected and analyzed for nutrient composition according to AOAC (2005) protocols [30]. The experiment lasted 28 days, covering the typical nursery feeding period commonly used in nutritional studies for nursery pigs [31,32]. Feed and water were provided without restriction during the experiment. The ambient temperature in the nursery room was kept at roughly 28 °C from d 1 to 14 and adjusted to 26 °C from d 15 to 28. All animal management procedures followed standard husbandry protocols.

### 2.2. Growth Performance

The pigs were weighed individually after a 12 h fast in the morning on d 1 and 14 in Exp. 1, and on d 1 and 28 in Exp. 2. Daily feed intake per replicate was recorded throughout the study to compute average daily feed intake (ADFI). Growth rate was expressed as average daily gain (ADG), calculated from the increase in body weight during the trial. The gain-to-feed ratio (G:F) was then determined by dividing weight gain by feed consumption [33]. The fecal score was assessed daily for each pig using a standardized scoring system, ranging from 1 to 5. A score of 1 indicated dry and tough feces; a score of 2 indicated firm feces that did not leave marks when picked up; a score of 3 represented soft feces that left marks when handled; a score of 4 referred to unshaped and sticky feces; and a score of 5 described watery feces typical of diarrhea [34].

$$Diarrhea\;occurrence\left(\%\right)=\frac{\mathrm{Number}\;\mathrm{of}\;\mathrm{Diarrhea}}{\mathrm{Number}\;\mathrm{of}\;\mathrm{Test}\;\mathrm{Pigs}\;\times \;\mathrm{Number}\;\mathrm{of}\;\mathrm{Test}\;\mathrm{Days}}\times 100$$

### 2.3. Sample Collection

On the last day of both studies, blood samples were collected from all pigs in Exp. 1 and 16 pigs in Exp. 2, 1 pig with average body weight from each pen, and 4 pigs from each treatment. The blood was sampled via the anterior vena cava using a 10 mL disposable syringe. A 2 mL portion of whole blood was collected into tubes containing ethylenediaminetetraacetic acid (EDTA) for complete blood count (CBC) testing. The residual blood was dispensed into tubes without anticoagulant, left to clot for 30 min at room temperature, and subsequently centrifuged at 2000× *g* for 15 min at 4 °C to isolate serum. The serum samples were preserved at −80 °C until further analysis.

Three of the four blood-sampled pigs from each treatment in Exp. 2 were selected and anesthetized, and then humanely slaughtered for intestinal histology analysis. Briefly, the small intestine was divided into three anatomical sections: the duodenum, jejunum, and ileum. From each section, tissue samples about 2 cm in length were collected. Each sample was gently washed with phosphate-buffered saline (PBS) to eliminate luminal contents, then fixed in 4% paraformaldehyde for 24 h for subsequent histological examination [35]. Colon contents were collected, frozen, and stored at −80 °C for microbiota assay.

### 2.4. Blood Profiles

A CBC assay was conducted promptly after blood collection in both Exp. 1 and Exp. 2. Whole blood samples were analyzed using an automated hematology analyzer (LH750, Beckman Coulter, Brea, CA, USA) following the operating instructions [13]. The parameters measured included white blood cell count (WBC), granulocyte ratio (GRA), monocyte percentage (MON), lymphocyte percentage (LYM), mean corpuscular hemoglobin (MCH), hemoglobin concentration (HGB), and mean corpuscular volume (MCV). Serum biochemical indicators, including albumin (ALB), total cholesterol (TC), total protein (TP), and total bilirubin (TBIL) from Exp. 2, were measured using an automatic biochemical analyzer (BC-30S, Myeri, Guangzhou, China).

### 2.5. Oxidative Stress and Immune Status

Malondialdehyde (MDA), glutathione peroxidase (GSH-Px), and superoxide dismutase (SOD) levels in Exp. 2 were measured using ELISA Kits (ml076370, ml026403, ml002400; Shanghai Enzyme-Linked Biotechnology Co., Ltd., Shanghai, China). All experimental procedures were carried out in accordance with the manufacturer’s protocols. Absorbance was measured at 550 nm for SOD, 412 nm for GSH-Px, and 532 nm for MDA. In addition, serum concentrations of tumor necrosis factor-α (TNF-α), interleukin-6 (IL-6), immunoglobulins A (IgA), and immunoglobulins G (IgG) were measured using specific ELISA kits (ml027109, ml002311, ml026837, and ml002328, Shanghai Enzyme-Linked Biotechnology Co., Shanghai, China), as described by Zhao et al. [36] and Moita et al. [37]. Optical density (OD) measurements were taken at wavelengths of 700, 340, and 450 nm by a microplate reader (Varioskan LUX, Thermo Fisher Scientific, Waltham, MA, USA). Concentrations were then determined by referencing the standard curves established for each assay.

### 2.6. Determination of Colonic Short-Chain Fatty Acids (SCFAs)

Approximately 1 g of colonic content from Exp. 2 was homogenized in 9 mL of ultrapure water and subjected to centrifugation at 12,000× *g* for 15 min at 4 °C. From the resulting supernatant, 1 mL was aliquoted and combined with 0.2 mL of metaphosphoric acid. The mixed supernatant samples were filtered through a 0.22 µm aqueous filter, and the mixture was centrifuged again at 12,000× *g* for 5 min. A volume of 1 µL of the final supernatant was promptly injected into a Trace 1300 gas chromatograph (Thermo Fisher Scientific, Waltham, MA, USA). The concentrations of acetic, propionic, isobutyric, butyric, isovaleric, and valeric acids were determined [13].

$$\mathrm{Concentration}\;\mathrm{of}\;\mathrm{SCFAs}=\frac{{\mathrm{Peak}\;\mathrm{area}}_{S,\;C}\;\times \;{\mathrm{Peak}\;\mathrm{area}}_{\mathrm{Std},\;A}\;\times \;{\mathrm{Standard}\;\mathrm{concentration}}_{C}}{{\mathrm{Peak}\;\mathrm{area}}_{S,\;A}\;\times \;{\mathrm{Peak}\;\mathrm{area}}_{\mathrm{Std},\;C}}$$

S: sample; C: certain acid; Std: standard; A: crotonic acid.

### 2.7. Detection of Intestinal Structure

Tissue samples from the duodenum, jejunum, and ileum in Exp. 2 were immersed in 4% paraformaldehyde and fixed for 24 h. The fixed tissues were then processed through a series of steps including dehydration, clearing, paraffin embedding, block trimming, sectioning, and mounting onto glass slides. Then, sections were stained with hematoxylin and eosin and sealed with neutral resin. Imaging was performed using the EVOS FL Auto Imaging System (Thermo Fisher Scientific, Waltham, MA, USA). Villus height (VH) and crypt depth (CD) were measured using Image-Pro Plus software (version 6.0, Media Cybernetics, Rockville, MD, USA), and the villus height-to-crypt depth ratio (V/C) was calculated accordingly.

### 2.8. Relative Abundance and Diversity of Colonic Microbiota

Microbial DNA was extracted from colonic content in Exp. 2 using an Omega Bio-tek DNA kit (Norcross, GA, USA). DNA concentration and purity were measured using a microspectrophotometer (Thermo Fisher Scientific, Waltham, MA, USA). The V3-V4 hypervariable region of the bacterial 16S rRNA gene was amplified using primers 338F338F (5′–ACTCCTACGGGGAGGCAGCAG–3′) and 806R (5′–GGACTACHVGGGTWTCTAAT–3′) on a T100 Thermal Cycler (Bio-Rad, Hercules, CA, USA). Amplicons were extracted from a 2% agarose gel and purified using the AxyPrep DNA Gel Extraction Kit (Axygen Biosciences, Union City, CA, USA). The purified amplicons were subjected to sequencing on an Illumina MiSeq PE300 platform (Illumina, San Diego, CA, USA) in paired-end mode.

Raw sequencing reads were filtered to remove low-quality bases (Q-score < 20) from the read tails using a 50 bp sliding window approach. Quality-filtered reads were denoised and dereplicated using the DADA2 plugin within the QIIME2 pipeline (version 2023.2), generating high-quality amplicon sequence variants (ASVs). Taxonomic assignment of these ASVs was conducted via the SILVA 16S rRNA gene database (version 138) using the QIIME2 feature-classifier plugin and BLAST2.15.0+-based alignment. Alpha diversity metrics, including Shannon diversity and Chao1 richness, were computed and statistically contrasted between groups via the Wilcoxon rank-sum test. Beta diversity was evaluated using Principal Coordinate Analysis (PCoA) based on Bray–Curtis dissimilarity to illustrate the similarity in microbial community composition across samples. The significance of variations in community structure was further assessed through PCoA clustering. Linear Discriminant Analysis Effect Size (LEfSe) was utilized to detect taxa with significantly different relative abundances at the genus level among treatment groups, in accordance with the approach described by Gormley et al. [38]. Additionally, Spearman correlation analysis was conducted to identify key bacterial taxa for constructing the co-occurrence network. Sequences classified as chloroplasts or mitochondria were excluded from downstream analyses to avoid host-derived contamination.

### 2.9. Colonic Metabolomic Analysis

Untargeted metabolomic analysis of colonic contents was performed using an ultra-high-performance liquid chromatography (UHPLC) system coupled with a mass spectrometer (Orbitrap Q Exactiv HF-X, Thermo Fisher Scientific Inc., Bremen, Germany). The raw data acquired via UHPLC-MS/MS were analyzed utilizing the Compound Discoverer software (version 3.1, Thermo Fisher Scientific Inc., Bremen, Germany). Peak alignment, deconvolution, and quantification of metabolite signals were carried out, and peak areas were extracted before integration of target ions. Molecular formulas were inferred from the peaks of molecular ions and fragment ions, and then compared against the mzCloud (https://www.mzcloud.org/), mzVault, and Masslist databases. Background signals were removed using blank sample controls, and metabolite intensities were normalized to obtain the final identification and quantitative data. The metabolite data from positive and negative ion modes were merged to create a comprehensive dataset for further analysis of target metabolites. To investigate potential functional relationships, Spearman correlation coefficients were calculated between the relative abundances of microbial species and identified metabolites. Correlation heatmaps were visualized using the R software package (version 1.8.4).

### 2.10. Statistical Analysis

Statistical analyses were conducted using a completely randomized design in SAS software (version 9.4, SAS Inc., Cary, NC, USA), with dietary treatment considered as a fixed effect and replicate as a random effect. For growth performance data, the experimental unit was the individual pig in Exp. 1 and the pen in Exp. 2. For all other measured parameters, individual pigs served as the experimental units [13]. To assess the dose–response effects of graded dietary niacinamide levels, orthogonal polynomial contrasts were utilized to evaluate linear and quadratic effects. For significant or trending effects, the NLMIXED procedure was used to determine optimal niacinamide supplementation levels [39,40,41]. The independent variable used in all models was daily niacinamide intake (mg/d), calculated by multiplying the dietary niacinamide concentration (mg/kg) by the ADFI of each replicate. Optimal intake values were then converted to corresponding dietary concentrations (mg/kg) by dividing by the ADFI. Statistical significance was set at *p* < 0.05, with trends noted for 0.05 ≤ *p* < 0.10.

## 3. Results

### 3.1. Growth Performance

In Exp. 1, increasing levels of dietary niacinamide quadratically increased (*p* < 0.05) BW on d 14, ADFI, ADG, and G:F (Table 3). Additionally, G:F tended to increase linearly (*p* = 0.05) with the rising niacinamide levels. Diarrhea occurrence decreased quadratically (*p* < 0.05) and showed a trend toward a linear reduction (*p* = 0.063). Niacinamide supplementation did not significantly affect fecal scores.

**Figure 1:**
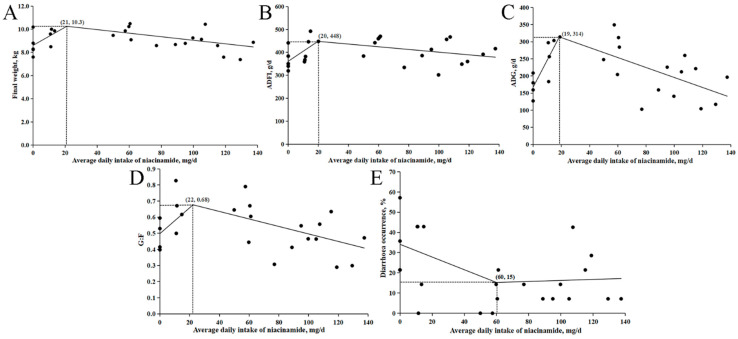
Broken-line regression models describing the relationship between daily niacinamide intake and growth indicators in nursery pigs in Exp. 1. (**A**) Body weight (BW) on d 14: y = 10.3 − 0.0784 × (21 − x) and y = 10.3 − 0.0153 × (x − 21); (**B**) Average daily feed intake (ADFI): y = 448 − 4.3926 × (20 − x) and y = 448 − 0.5875 × (x − 20); (**C**) Average daily gain (ADG): y = 314 − 7.9033 × (19 − x) and y = 314 − 1.4562 × (x − 19); (**D**) Gain-to-feed ratio (G:F): y = 0.68 − 0.0080 × (22 − x) and y = 0.68 − 0.0023 × (x − 22); (**E**) Diarrhea incidence (%): y = 15.2 + 0.3146 × (60 − x) and y = 15.2 + 0.0251 × (x − 60). In all models, x represents daily niacinamide intake (mg/d), and y represents the corresponding response variable. *p*-values for all overall models, slopes, and breakpoints were <0.05.

The relationship between these response variables and daily niacinamide intake is shown in Figure 1. The estimated optimal intakes for achieving maximum BW on d 14, ADFI, ADG, G:F, and minimum diarrhea occurrence were 21, 20, 19, 22, and 60 mg/d, respectively. Based on the predicted ADFI values from the intake–response model (Figure 1B), these optimal intakes correspond to approximate dietary concentrations of 47, 43, 45, 49, and 140 mg/kg of niacinamide.

In Exp. 2, the G:F increased linearly (*p* < 0.05) and showed a quadratic trend (*p* = 0.082) with increasing dietary niacinamide levels in nursery pigs (Table 4). Fecal scores decreased linearly (*p* < 0.05) with increasing niacinamide levels. Diarrhea occurrence decreased (*p* < 0.05) linearly and quadratically with increasing niacinamide levels.

**Figure 2:**
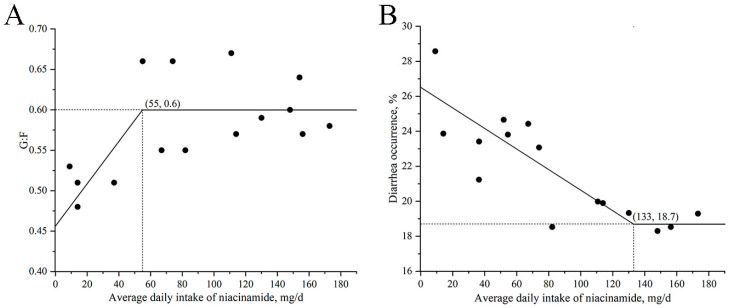
Broken-line regression models describing the relationship between daily niacinamide intake and gain-to-feed ratio (G:F) and diarrhea incidence in nursery pigs in Exp. 2. (**A**) G:F = 0.60 − 0.0027 × (55 − x, mg/d), for x < 55 mg/d and y = 0.6 for x ≥ 55 mg/d. (**B**) Diarrhea occurrence = 18.7 − 0.0568 × (133 − x), for x < 133 mg/d and y = 18.7 for x ≥ 133 mg/d. In all models, x represents daily niacinamide intake (mg/d), and y represents the corresponding response variable. *p*-values for all overall models, slopes, and breakpoints were <0.05.

The relationship between daily niacinamide intake and both G:F and diarrhea occurrence is shown in Figure 2. The maximum G:F was observed at a daily intake of 55 mg, corresponding to an estimated dietary concentration of 130 mg/kg niacinamide. Meanwhile, the lowest diarrhea occurrence was at a daily intake of 133 mg/d, equivalent to approximately 314 mg/kg of dietary niacinamide. As ADFI did not differ significantly among treatments in Exp. 2, the estimated dietary niacinamide concentrations were calculated based on the overall average feed intake (424 g/d) across all groups rather than derived from regression models.

### 3.2. Blood Profiles

In Exp. 1, the percentage of MON, HGB, and MCV showed quadratic responses (*p* < 0.05) with increasing levels of niacinamide (Table 5). No significant differences were observed in other hematological parameters.

**Figure 3:**
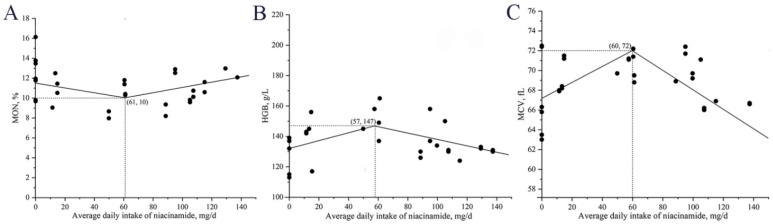
Broken-line regression models describing the relationship between daily niacinamide intake and blood parameters in nursery pigs in Exp. 1. (**A**) monocytes (MON): y = 10.4 + 0.0242 × (61 − x) and y = 10.4 + 0.0265 × (x − 61); (**B**) hemoglobin (HGB): y = 148 − 0.2561 × (58 − x) and y = 148 − 0.2111 × (x − 58); (**C**) mean erythrocyte volume (MCV): y = 72.0 − 0.0810 × (60 − x) and y = 72.0 − 0.0982 × (x − 60). In all models, x represents daily niacinamide intake (mg/d), and y represents the corresponding response variable. *p*-values for all overall models, slopes, and breakpoints were <0.05.

The relationship between daily niacinamide intake and MON, HGB, and MCV are illustrated in Figure 3. The minimum MON was observed at a daily intake of 61 mg, the maximum HGB was observed at a daily intake of 57 mg, the maximum MCV was observed at a daily intake of 60 mg, corresponding to an estimated dietary concentration of approximately 140 mg/kg niacinamide.

In Exp. 2, the percentage of HGB and IgA increased quadratically (*p* < 0.05) in response to niacinamide supplementation (Table 6). No adverse effects on other serum biochemical parameters, antioxidant enzyme activities, or immune-related indicators were observed in nursery pigs, indicating that dietary niacinamide was well tolerated at the tested levels.

**Figure 4:**
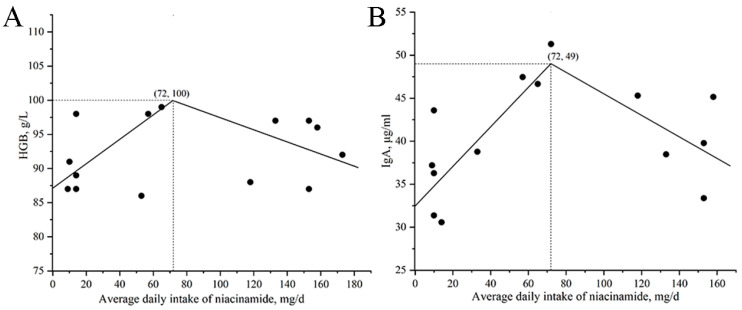
Broken-line regression models describing the relationship between daily niacinamide intake and hemoglobin (HGB) and immunoglobulin A (IgA) in nursery pigs in Exp. 2. (**A**) HGB: y = 101 − 0.1748 × (72 − x) and y = 101 − 0.0880 × (x − 72); (**B**) IgA: y = 49.4 − 0.2303 × (72 − x) and y = 49.4 − 0.1249 × (x − 72). In all models, x represents daily niacinamide intake (mg/d) and y represents the corresponding response variable. *p*-values for all overall models, slopes, and breakpoints were <0.05.

The relationship between daily niacinamide intake and both HGB and IgA is shown in Figure 4. The maximum HGB and IgA were observed at a daily intake of 72 mg, corresponding to an estimated dietary niacinamide concentration of 170 mg/kg.

### 3.3. Intestinal Morphology

In Exp. 2, VH in the duodenum increased both linearly and quadratically (*p* < 0.05) with rising dietary levels of niacinamide (Table 7). In contrast, the V/C in the duodenum decreased linearly (*p* < 0.05). In the jejunum, VH increased linearly (*p* < 0.05) and exhibited a quadratic trend (*p* = 0.094) with increasing niacinamide supplementation. In the ileum, both CD and the V/C ratio responded quadratically (*p* < 0.05) to increasing dietary niacinamide levels.

**Figure 5:**
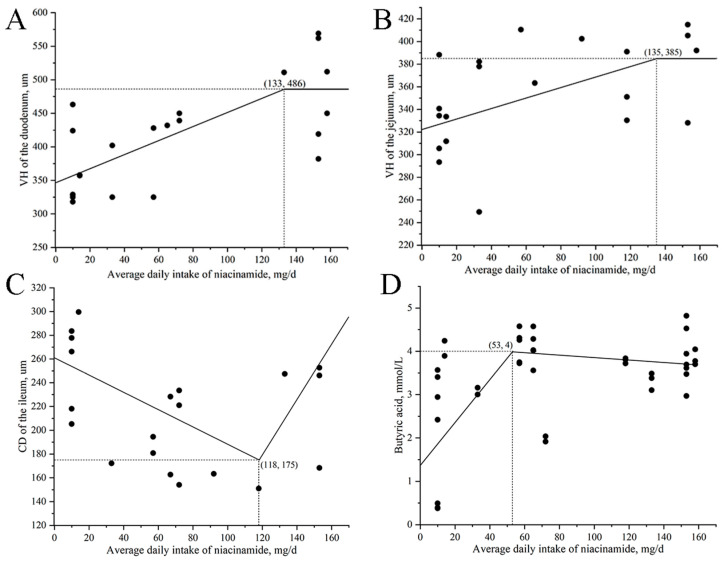
Broken-line regression models describing the relationship between daily niacinamide intake and intestine morphology indicators and butyric acid concentrations in nursery pigs in Exp. 2. (**A**) Villus height (VH) of the duodenum = 133 − 1.0480 × (486 − x), for x < 133 mg/d and y = 486 for x ≥ 133 mg/d. (**B**) VH of the jejunum = 135 − 0.4644 × (385 − x), for x < 135 mg/d and y = 385 for x ≥ 135 mg/d. (**C**) Crypt depth (CD) of the ileum: y = 175 + 0.7286 × (118 − x) and y = 175 + 2.3327 × (x − 118); (**D**) Butyric acid: y = 4.1 − 0.0495 × (53.2 − x) and y = 4.1 − 0.0028 × (x − 53.2). In all models, x represents daily niacinamide intake (mg/d), and y represents the corresponding response variable. *p*-values for all overall models, slopes, and breakpoints were <0.05.

The relationship between daily niacinamide intake and VH of the duodenum and jejunum, as well as CD of the ileum, is shown in Figure 5A–C. The maximum VH of the duodenum and jejunum was observed at daily intakes of 133 mg and 135 mg, respectively, corresponding to an estimated dietary concentration of approximately 315 mg/kg niacinamide. The minimum CD of the ileum was observed at a daily intake of 118 mg, corresponding to approximately 280 mg/kg niacinamide.

### 3.4. Colonic SCFAs

The colonic concentrations of TVFA and acetic acid increased linearly (*p* < 0.05) with rising levels of dietary niacinamide (Table 8). Butyric acid concentration also exhibited a linear increase (*p* < 0.05), along with a tendency for a quadratic response (*p* = 0.063). The relationship between daily niacinamide intake and butyric acid concentration is shown in Figure 5D. The maximum value of butyric acid was observed at a daily intake of 53 mg, corresponding to an estimated dietary concentration of 125 mg/kg niacinamide.

### 3.5. Relative Abundance and Diversity of Colonic Microbiota

**Figure 6:**
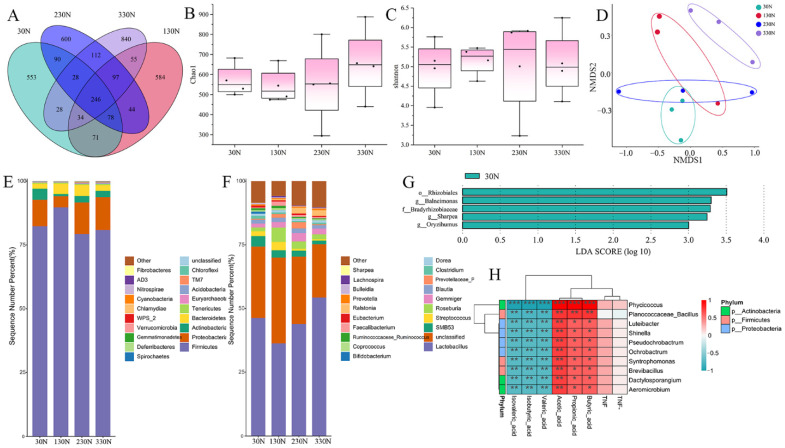
Effect of dietary supplementation with different levels of niacinamide on colonic microorganisms in nursery pigs in Exp. 2. (**A**) Venn analysis at the ASV level; (**B**) Chao 1 index of colonic microbiota; (**C**) Shannon index of colonic microbiota; (**D**) analysis of β-diversity of colonic microbial composition; (**E**) relative abundance on phylum level between the four groups; (**F**) relative abundance on genus level between the four groups; (**G**) statistical analysis of differential species of abundance; (**H**) correlation analysis of abundance of top 10 bacterial genera with colonic short-chain fatty acid correlation. 30N, diet containing 30 mg/kg niacinamide supplementation; 130N, diet containing 130 mg/kg niacinamide supplementation; 230N, diet containing 230 mg/kg niacinamide supplementation; 330N, diet containing 330 mg/kg niacinamide supplementation. * 0.01 < *p* ≤ 0.05, ** 0.001 < *p* ≤ 0.01, *** *p* ≤ 0.001.

The colonic contents of the four groups detected 1128, 1209, 1295, and 1440 ASVs, respectively. The four groups shared 246 ASVs, whereas unique ASVs were 553, 584, 600, and 840 for the 30N, 130N, 230N, and 330N groups, respectively (Figure 6A).

The alpha diversity of colonic microbiota was evaluated using the Shannon and Chao1 indices. No significant variation in colonic microbial alpha diversity was observed among the four groups of nursery pigs (Figure 6B,C).

The effect on the beta diversity of colonic microorganisms in nursery pigs was analyzed. The results of PCoA analysis (PC1 = 16.66%; PC2 = 14.31%) showed that there were no significant differences in ASVs of colonic microbiota in the 30N, 130N, and 230N groups. However, the 330N group was clearly separated from the other three groups (Figure 6D).

Differences in the proportional composition of colonic microbiota at both phylum and genus taxonomic levels were examined across the four diet groups (Figure 6E,F). Increasing dietary niacinamide levels altered the microbial composition of the colon. At the phylum level, Firmicutes and Proteobacteria were dominant across all groups, while other relatively abundant phyla included Actinobacteria, Bacteroidetes, Stramenobacteria, Euryarchaeota, Chlamydomonas, and Chloroflexi. The relative abundance of Firmicutes was higher (*p* < 0.05) in the 130N group compared with the other groups. Conversely, the abundance of Actinobacterium was lower (*p* < 0.05) in the 130N group. At the genus level, the proportion of *Lactobacillus* was higher (*p* < 0.05) in the 330N group compared with the other groups. In contrast, the proportion of *SMB53* was the lowest (*p* < 0.05) in the 330N group. Additionally, the abundance of *Streptococcus* was reduced (*p* < 0.05) in the 230N and 330N groups relative to other groups.

LEfSe analysis of pig colonic microorganisms in four groups of nursery pigs (LDA > 2.0, Figure 6G) revealed that only the 30N group had specific dominant genera, and they were o_Rhizobiales, g_*Balneimonas*, f_Bradyrhizobiaceae, g_*Sharpea*, and g_*Oryzihumus*.

To explore the relationship between colonic SCFAs and intestinal microbiota, Spearman correlation analysis was performed between the top 10 most abundant bacterial genera and colonic SCFAs concentrations in nursery pigs (Figure 6H). The results showed that isovaleric acid, isobutyric acid, and valeric acid were negatively correlated (*p* < 0.05) with the relative abundance of *Phycicoccus*, *Luteibacter*, *Shinella*, *Pseudochrobactrum*, *Ochrobactrum*, *Syntrophomonas*, *Brevibacillus*, *Dactylosporangium*, *Aeromicrobium*, and *Planococcaceae_Bacillus*. In contrast, acetic acid, butyric acid, and propionic acid were positively correlated (*p* < 0.05) with the same genera, suggesting that these bacteria may play differential roles in SCFA production or metabolism.

### 3.6. Metabolomic Analysis of Colonic Tissue

**Figure 7:**
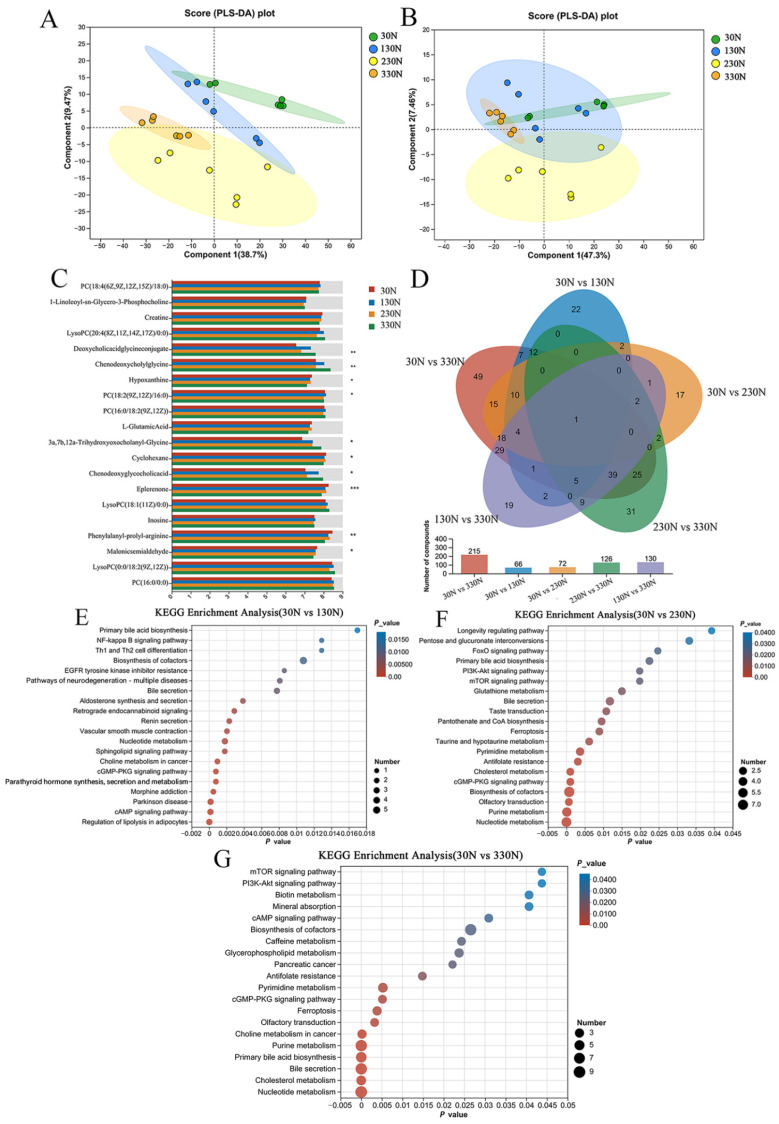
Effect of dietary addition of different levels of niacinamide on colonic metabolites in nursery pigs in Exp. 2. (**A**) Partial Least Squares Discriminant Analysis (PLS-DA) between groups Cation mode; (**B**) Partial Least Squares Discriminant Analysis (PLS-DA) between groups Anion mode; (**C**) Comparison of multiple groups of differential metabolites; (**D**) Metabolites Comparison of Differential Metabolites Between the Two Groups Venn Diagrams; (**E**) Bubble plot of KEGG enrichment analysis in 30N group vs. 130N group; (**F**) Bubble plot of KEGG enrichment analysis in 30N group vs. 230N group; (**G**) Bubble plot of KEGG enrichment analysis in 30N group vs. 330N group. 30N, diet containing 30 mg/kg niacinamide supplementation; 130N, diet containing 130 mg/kg niacinamide supplementation; 230N, diet containing 230 mg/kg niacinamide supplementation; 330N, diet containing 330 mg/kg niacinamide supplementation. * 0.01 < *p* ≤ 0.05, ** 0.001 < *p* ≤ 0.01, *** *p* ≤ 0.001.

Untargeted metabolomic profiling of colonic tissue was conducted to elucidate further the potential metabolic pathways through which intestinal microbiota may influence growth performance in nursery pigs. Metabolites detected in both positive and negative ionization modes were combined to construct a comprehensive metabolite dataset. Partial least squares discriminant analysis (PLS-DA) was executed to determine intergroup differentiation. As shown in Figure 7A,B, clear separation was observed among the 30N, 130N, 230N, and 330N treatment groups in the positive ion mode. The R^2^Y and Q^2^ values for the comparative models were close to 1, indicating strong model fitness and predictive capability, with no signs of overfitting.

Figure 7C compares the differential abundance of key metabolites in the 30N, 130N, 230N, and 330N groups. The relative abundances of deoxycholic acid glycine conjugate and 3a, 7b, 12a-trihydroxyoxocholanyl-glycine were higher (*p* < 0.01) in group 330N than in group 30N. Chenodeoxycholylglycine, hypoxanthine, and chenodeoxyglycocholie acid (goose deoxycholic acid) were higher (*p* < 0.05) in the 330N group than in the 30N and 230N groups. In contrast, the relative abundances of hypoxanthine, cyclohexane, malonic semialdehyde, eplerenone, and phenylalanyl-prolyl-arginine were lower (*p* < 0.05) in group 330N than those of the 30N group.

Differential metabolites were identified using univariate analysis based on fold change (FC) and Student’s t-test. Metabolites were considered significantly different if they met the criteria of FC > 1.5 and *p* < 0.05. As shown in Figure 7D, a total of 66, 72, and 215 differential metabolites were identified in the 30N group when compared to the 130N, 230N, and 330N groups, respectively. Additionally, 100 differential metabolites were identified between the 130N and 330N groups, and 126 between the 230N and 330N groups.

KEGG pathway enrichment analysis revealed distinct metabolic alterations across treatment groups relative to the 30N group. As shown in Figure 7E, the 130N group exhibited enrichment (*p* < 0.05) in pathways related to renin secretion, nucleotide metabolism, cGMP–PKG signaling, and cAMP signaling. In the 230N group, enriched pathways (*p* < 0.05) primarily included pyrimidine metabolism, cGMP-PKG signaling, biosynthesis of cofactors, purine metabolism, and nucleotide metabolism (Figure 7F). Compared to the 30N group, the 330N group showed enrichment (*p* < 0.05) in multiple pathways, including pyrimidine metabolism, cGMP-PKG signaling, ferroptosis, olfactory transduction, purine metabolism, primary bile acid biosynthesis, cholesterol metabolism, and nucleotide metabolism (Figure 7G).

## 4. Discussion

This research offers novel perspectives on the functional demand for dietary niacinamide in nursery pigs consuming low-protein diets supplemented with or without pharmacological ZnO [42]. Although niacin is known to support energy metabolism, redox regulation, and epithelial integrity [11,12], its optimal inclusion level under practical feeding strategies remains unclear. Our findings demonstrate that nursery pigs responded to niacinamide in a dose-dependent manner and that the requirement increased in the absence of ZnO. Moreover, the beneficial effects of niacinamide not only improved intestinal morphology, microbial composition, fermentation products, systemic immunity, and host metabolism but also promoted growth performance. These findings support our hypothesis that niacinamide contributes to maintaining intestinal function and physiological homeostasis in nursery pigs, particularly under the current dietary Zn level. The following sections discuss these effects in detail, with a focus on dose-dependent responses across intestinal morphology, microbial composition, fermentation products, metabolic pathways, and systemic biomarkers, as well as growth performance. To compensate for the limited number of replicates, we have verified that consistent results were obtained from the two experiments in growth performance and blood indices, whether the pigs were fed individually or in groups. These results appeared to be supported by the antioxidant markers and intestinal morphology measured in Exp. 2.

Beneficial responses of weaned pigs in the growth performance to dietary supplementation of niacinamide were observed in both experiments. However, the response patterns to the dose of the supplementation and the estimated optimal levels for niacinamide from pigs with supplemented dietary ZnO were different from those without supplemented ZnO. When ZnO was included in the low-protein diets, BW, ADG, ADFI, and G:F increased quadratically, with peak responses observed at approximately 50 mg/kg of niacinamide. These results suggest that the niacinamide level supplemented to a low-protein diet for optimal growth performance in nursery pigs is associated with the dietary Zn level. This is probably due to the negative impact of a low-protein diet on Zn absorption and efficiency [23,24]. Indeed, the G:F was improved linearly with the increase in niacinamide supplementation, with the highest efficiency observed at 130 mg/kg, in Exp. 2 with no ZnO addition. Moreover, the diarrhea occurrence decreased progressively with an increase in niacinamide, reaching a lowest level at approximately 315 mg/kg. These findings indicate that, with no supplementation of ZnO, higher levels of niacinamide are required to sustain feed efficiency and intestinal stability. According to Wu et al., supplementing 360 mg/kg niacinamide in low-protein diets reduced fecal, urinary, and total nitrogen excretion without impairing nitrogen retention or ADG in growing pigs (BW = 40 kg) [43]. Similarly, Feng et al. observed that feeding 40 mg/d of niacin for three days effectively attenuated the rate of weight loss and diarrhea in weaned piglets under starvation stress [44]. Collectively, the dose–response curves from both experiments and results reported in previous studies suggest that the dietary requirement of niacinamide is affected by dietary protein level and the growth stage of the animal, in which the dietary composition, especially the availability of functional compounds such as Zn, may play an important role. These findings highlight the importance of evaluating the effect of Zn availability on current niacin recommendations under low-protein diets.

The observed improvements in growth performance were accompanied by positive changes in indicators of intestinal health, suggesting that niacinamide’s beneficial effects are closely linked to its role in maintaining intestinal function. During the early post-weaning period, pigs often experience impaired intestinal structure, microbial imbalance, and immune disruption, all of which compromise nutrient utilization and growth efficiency [45,46]. Therefore, strategies that promote intestinal development and stabilize the intestinal environment are essential for supporting performance under weaning stress.

Previous studies have shown that niacinamide supplementation contributes to intestinal barrier integrity, epithelial regeneration, and mucosal immunity in pigs [13,14,15,47]. Consistently, the present study demonstrated that niacinamide reduced diarrhea scores, improved intestinal morphology, enriched beneficial microbial taxa, and modulated microbial fermentation. These coordinated improvements suggest that the intestine is a primary site of niacinamide action, particularly under conditions with no ZnO supplementation, where intestinal health is more vulnerable.

Niacinamide supplementation reduced diarrhea severity in both experiments, showing a clear dose-dependent pattern. In Exp. 1, conducted under ZnO-supplemented conditions, diarrhea occurrence decreased progressively, reaching the lowest levels at approximately 140 mg/kg. In contrast, under ZnO-free conditions in Exp. 2, diarrhea occurrence linearly reduced throughout the whole tested period, with a minimum at around 315 mg/kg. These findings indicate that higher levels of niacinamide are needed to stabilize intestinal function in the absence of ZnO supplementation. The reduction in diarrhea could be attributed to the improved mucosal barrier function, enhanced microbial stability, or modulation of local immune responses, all of which are known targets of niacinamide action [48,49]. Although some of these parameters were evaluated in this study, further studies are warranted to elucidate the underlying mechanisms. Similar effects were reported in previous studies, where dietary niacin supplementation linearly decreased fecal scores and intestinal inflammation in weaned pigs [43,47] and the incidence of diarrhea under protein-restricted conditions from high-level (330 mg/kg) compared to the low-level niacinamide (30 mg/kg) supplementation [23]. These findings highlight the protective role of niacinamide in maintaining intestinal stability and suggest that post-weaning diarrhea severity may serve as a sensitive indicator of the functional requirement for this vitamin across different dietary regimens.

Supplementation with 315 mg/kg of nicotinamide maximized VH in the duodenum and jejunum, while 280 mg/kg minimized CD in the ileum, suggesting that adding an appropriate amount of nicotinamide enhances intestinal absorptive surface area and promotes intestinal maturation. These findings are consistent with the reports in previous studies, demonstrating that niacin or its derivatives support epithelial turnover and preserve mucosal barrier integrity [40,50]. For instance, dietary niacin linearly increased jejunal VH, CD, and villus width in weaned pigs at 25 days of age. It promoted the expression of key genes involved in nutrient transport and epithelial integrity, including SLC5A1, SLC15A1, SLC6A19, and tight junction proteins such as occludin and claudin-1 after 14 days of feeding a diet with niacin supplementation [50]. These effects may be linked to niacinamide’s role in regulating epithelial barrier function, electrolyte transport, and fluid absorption. Taken together, these results suggest that niacinamide supplementation benefits weaned pigs with post-weaning intestinal development and may serve as an effective nutritional strategy to enhance mucosal adaptation, particularly using a low-protein diet with no Zn supplementation.

Niacinamide supplementation altered the composition of the intestinal microbiota, characterized by a heightened proportional prevalence of *Lactobacillus* and reduced abundance of *Streptococcus* in the 330N group. These microbial shifts are considered beneficial for maintaining intestinal homeostasis. *Lactobacillus* species are known to enhance epithelial function, produce organic acids, and inhibit pathogenic bacteria [51,52]. In contrast, elevated levels of *Streptococcus* have been associated with intestinal inflammation and post-weaning diarrhea in pigs [53,54]. These microbial shifts might contribute to the observed improvements in villus structure and reduction in diarrhea severity. Consistent with our findings, dietary niacin influences microbial structure and function in the intestine, which has also been reported in previous studies [47,55]. For example, Liu et al. [47] found that niacin supplementation elevated the proportional prevalence of *Lactobacillus* and *Dorea*, whereas it diminished the proportional prevalence of *Peptococcus* in the colon, compared with a multi-probiotic product. Collectively, these results suggest that niacinamide promotes a more favorable microbial community, thereby enhancing intestinal resilience during the critical weaning period.

In addition to microbial shifts, niacinamide modulated colonic fermentation activity, as reflected by changes in SCFAs profiles. Acetate concentrations increased linearly with the increased niacinamide levels, while butyrate peaked at 125 mg/kg and did not increase further at higher doses. The rise in acetate may be attributed to the expansion of acetate-producing bacteria such as *Lactobacillus*, which were enriched in response to niacinamide [51,52]. Acetate plays critical roles in epithelial barrier maintenance, anti-inflammatory signaling, and serves as an energy substrate for peripheral tissues [56]. The consistent rise in acetate suggests a more active and stable microbial fermentation pattern with higher niacinamide intake. In contrast, the non-linear response of butyrate may be due to the limited responsiveness of key butyrate-producing taxa, primarily obligate anaerobes such as *Ruminococcaceae* and *Lachnospiraceae*, to niacinamide [57,58]. Additionally, enhanced villus development could accelerate butyrate uptake by colonocytes, limiting its accumulation in luminal contents despite continued production. Given the well-established roles of SCFAs in maintaining intestinal integrity and immune regulation, these fermentation changes likely contributed to the suggested improvement in intestinal morphology and reduced diarrhea in this study.

Metabolomic profiling of colonic tissue revealed that niacinamide supplementation influenced several host metabolic pathways, including bile acid metabolism and purine metabolism in the 330N group. These changes indicate that niacinamide not only acts as a dietary precursor of NAD^+^ biosynthesis but also modulates host-microbe metabolic crosstalk at the intestinal level [59]. Specifically, glycocholic acid and taurocholic acid concentrations increased in a dose-dependent manner, while chenodeoxyglycocholic acid was elevated in the 330N compared to the 30N and 230N groups. KEGG pathway enrichment analysis further confirmed the upregulation of primary bile acid biosynthesis in this group. Bile acids are known to exert direct antimicrobial effects and influence microbial composition through signaling pathways such as Takeda G-protein-coupled receptor 5 (TGR5) and farnesoid X receptor (FXR) activation [60]. Moreover, bile salt hydrolase activity, common in *Bacteroides*, *Eubacterium*, and *Lactobacillus*, promotes deconjugation of bile acids, increasing their hydrophobicity and antimicrobial potency [61]. These effects may underline the observed enrichment of *Lactobacillus* and suppression of *Streptococcus* in high-niacinamide groups. Additionally, enhanced purine metabolism may support epithelial renewal, as purine derivatives such as hypoxanthine and guanine are essential for DNA synthesis in rapidly proliferating intestinal cells [62,63]. These findings suggest that dietary niacinamide modulates both microbial activity and host epithelial metabolism through multiple metabolic pathways, thereby contributing to improved intestinal health.

Beyond local intestinal effects, niacinamide supplementation also altered several systemic blood biomarkers associated with immune regulation and nutritional status. In Exp. 1, monocyte counts increased quadratically, peaking at 140 mg/kg, suggesting enhanced innate immune responses. In Exp. 2, serum IgA concentrations increased with niacinamide supplementation, reaching the highest level at 170 mg/kg. As a key component of humoral immunity, IgA binds to antigens and helps modulate immune responses [64]. The increase in IgA indicates that niacinamide may support B-cell maturation and contribute to immune homeostasis during the post-weaning period [65]. Hematological indicators related to oxygen transport also responded positively. Hemoglobin concentrations increased quadratically in both experiments, with the peak at 140 mg/kg for the number of monocytes in Exp. 1 and 170 mg/kg for serum IgA in Exp. 2. Mean corpuscular volume also showed a quadratic increase, reaching its maximum at 140 mg/kg in Exp. 1. These improvements suggest more efficient oxygen delivery and energy metabolism, which are critical for sustaining growth in nursery pigs [66]. The increase in effective dose required for a diet with no ZnO supplementation confirmed that Zn availability is important for the efficiency of niacinamide supplementation in the diet of nursery pigs, especially in the low-protein diet.

In summary, the improvements in growth performance observed in this study can be attributed to coordinated enhancements in intestinal health, optimization in microbial ecology, and homeostasis in systemic physiological function. These beneficiaries of niacinamide supplementation are supported by the reduced diarrhea, which tended to improve villus morphology, increased beneficial bacteria such as *Lactobacillus*, and promoted the production of SCFAs, particularly acetate and butyrate, in this study. These changes fully explain that niacinamide supplementation promotes a more stable and functional intestinal environment. In addition, results from the metabolomic analysis of colonic tissue revealed that niacinamide upregulated bile acid and purine metabolic pathways, thereby enhancing host–microbiota interactions and epithelial renewal. Furthermore, increased HGB, MCV, and IgA levels in circulation further reflected the improvements in nutrient delivery and immune competence. Therefore, these outcomes indicate that niacinamide supports nursery pig growth through multiple interrelated mechanisms.

The different peak responses obtained from the two experiments suggest that the supplemental level of niacinamide in low-protein diets for optimal benefits will be affected by dietary Zn level. Because protein is a major source of dietary Zn and the absorption of Zn is associated with dietary protein level and Zn availability [23,24], the presence of ZnO in the diet increased Zn availability, improved intestinal stability, and reduced weaning stress, thereby reducing the amount of niacinamide required for the peak responses. The negative impact of a low-protein diet on Zn absorption and utilization was also recently reported in broiler chicks [67]. Therefore, these findings highlight that dietary supplementation of niacinamide for optimal benefits is affected by other nutrients in the diet. Current nutritional recommendations may underestimate niacin needs for optimizing performance in nursery pigs, particularly the use of a low-protein diet, in which Zn may play an important role in compensatory functions and mechanisms of niacinamide.

## 5. Conclusions

This study demonstrates that supplementation of niacinamide promotes growth performance, intestinal integrity, and systemic health in nursery pigs fed with low-protein diets. The supplemental dose required for optimal promotion is associated with Zn availability. The findings provide new insight into the functional requirement of niacinamide in low-protein nutrition systems, and further studies are needed to elucidate its underlying mechanisms, especially the role of Zn availability and source.

## Figures and Tables

**Table 1:** Composition and nutrient levels of experimental diets (%, as-fed basis, Exp. 1).

Item	Dietary Composition
Ingredients	
Corn	65.50
Whey powder	10.00
Extruded soybean	3.18
Fermented soybean meal	5.00
Soybean protein concentrate	5.00
Fish meal	5.00
Soybean oil	0.18
L-Lys HCl (78%)	0.84
DL-Met (99%)	0.33
L-Thr (98.5%)	0.38
L-Trp (98%)	0.15
L-Val (98%)	0.27
Limestone	0.96
Monocalcium phosphate	0.40
ZnO	0.20
NaCl	0.71
Premix ^1^	1.90
Analyzed composition	
Dry matter	89.43
Crude protein	17.47
Ether extract	3.67
Neutral detergent fiber	9.22
Ca	0.69
Total P	0.61
Calculated composition	
NE, kcal/kg	2550
SID ^2^ Lys	1.40
SID Met + Cys	0.90
SID Thr	0.97
SID Trp	0.30

^1^ Per kilogram of the complete diet contained the following components: 0, 30, 130, 230, or 330 mg of niacinamide (in line with the respective treatment groups); 10,000 IU of vitamin A from vitamin A acetate; 1900 IU of vitamin D_3_; 50 IU of vitamin E; 6 mg of vitamin K_3_; 4 mg of vitamin B_1_; 9 mg of vitamin B_2_; 15 mg of calcium pantothenate; 0.20 mg of biotin; 0.03 mg of vitamin B_12_; 40 mg of Cu from copper sulfate; 70 mg of Fe from ferrous sulfate; 35 mg of Mn from manganous oxide; 0.50 mg of I from potassium iodide; and 0.30 mg of Se from sodium selenite. ^2^ SID, standardized ileal digestibility.

**Table 2:** Composition and nutrient levels of experimental diets (%, as-fed basis, Exp. 2).

Item	Diet
Ingredients	
Corn	61.32
Extruded soybean	12.00
Fermented soybean meal	11.00
Whey powder	7.00
Fish meal	3.00
Soybean oil	0.08
L-Lys HCl (78%)	0.64
DL-Met (99%)	0.28
L-Thr (98.5%)	0.27
L-Trp (98%)	0.09
Limestone	0.46
Monocalcium phosphate	1.32
NaCl	0.54
Premix ^1^	2.00
Analyzed composition	
Dry matter	87.76
Crude protein	17.41
Ether extract	4.42
Neutral detergent fiber	8.74
Ca	0.68
Total P	0.59
Calculated composition	
NE, kcal/kg	2500
SID ^2^ Lys	1.20
SID Met + Cys	0.73
SID Thr	0.79
SID Trp	0.25

^1^ Per kilogram of the complete diet contained the following components: 0, 30, 130, 230, or 330 mg of niacinamide (in line with the respective treatment groups); 10,000 IU of vitamin A; 1900 IU of vitamin D_3_; 50 IU of vitamin E; 6 mg of vitamin K_3_; 4 mg of vitamin B_1_; 9 mg of vitamin B_2_; 15 mg of calcium pantothenate; 0.20 mg of biotin; 0.03 mg of vitamin B_12_; 40 mg of Cu; 70 mg of Fe; 35 mg of Mn; 0.50 mg of I; and 0.30 mg of Se. ^2^ SID, standardized ileal digestibility.

**Table 3:** Growth response of nursery pigs to diets containing varying amounts of niacinamide (Exp. 1) ^1^.

Items	Treatments					SEM	*p*-Value	
	0NAM	30NAM	130NAM	230NAM	330NAM		Linear	Quad
BW d 1, kg	6.1	6.1	5.9	6.4	6.2	0.1	0.587	0.862
BW d 14, kg	8.6	10.0	9.8	9.1	8.3	0.2	0.506	0.007
ADFI, g/d	367	410	444	412	364	11	0.698	0.009
ADG, g/d	184	277	280	192	156	14	0.128	0.001
G:F	0.50	0.68	0.63	0.46	0.43	0.03	0.050	0.005
Fecal score	3.56	3.44	2.95	3.33	3.17	0.09	0.180	0.184
Diarrhea occurrence	31.4	28.6	8.6	15.7	15.7	5.97	0.063	0.038

^1^ 0NAM: experimental diet without niacinamide; 30NAM: diet supplemented with 30 mg/kg niacinamide; 130NAM: diet supplemented with 130 mg/kg niacinamide; 230NAM: diet supplemented with 230 mg/kg niacinamide; 330NAM: diet supplemented with 330 mg/kg niacinamide. *p*-value: *p* < 0.05 indicating significant differences and 0.05 ≤ *p* < 0.10 meaning a trend; Quad, quadratic; ADFI, average daily feed intake; ADG, average daily gain; G:F, gain-to-feed ratio; SEM, standard error of the means (*n* = 6).

**Table 4:** Growth response of nursery pigs to diets containing varying amounts of niacinamide (Exp. 2) ^1^.

Items	Treatments				SEM	*p*-Value	
	30N	130N	230N	330N		Linear	Quad
BW d 1, kg	7.8	7.9	8.0	7.9	0.3	0.971	0.999
BW d 28, kg	13.8	14.5	15.6	15.0	0.7	0.547	0.734
ADFI, g/d	410	414	433	437	28	0.854	0.822
ADG, g/d	209	236	272	253	20	0.387	0.533
G:F	0.51	0.57	0.63	0.58	0.02	0.041	0.082
Fecal score	2.98	2.93	2.76	2.55	0.07	0.028	0.557
Diarrhea occurrence	26.2	23.2	21.1	19.1	0.8	0.001	0.001

^1^ 30N: experimental basal diet supplemented with 30 mg/kg niacinamide; 130N: diet supplemented with 130 mg/kg niacinamide; 230N: diet supplemented with 230 mg/kg niacinamide; 330N: diet supplemented with 330 mg/kg niacinamide. Quad, quadratic; ADFI, average daily feed intake; ADG, average daily gain; G:F, gain-to-feed ratio; SEM, standard error of the means (*n* = 4). *p*-value: *p* < 0.05 indicating significant differences and 0.05 ≤ *p* < 0.10 meaning a trend.

**Table 5:** Serum physiological parameters and biochemical analyses of nursery pigs fed diets with increasing levels of niacinamide (Exp. 1) ^1^.

Items	Treatments					SEM	*p*-Value	
	0NAM	30NAM	130NAM	230NAM	330NAM		Linear	Quad
WBC, ×10^9^/L	12.6	14.0	8.6	13.5	10.4	3.0	0.157	0.353
LYM, %	21.2	16.6	24.0	20.8	21.0	6.9	0.627	0.886
MON, %	12.2	10.3	10.7	10.4	13.0	0.39	0.658	0.003
GRA, %	63.7	71.4	61.3	66.8	63.9	1.2	0.453	0.733
HGB, g/L	130	140	142	146	134	3	0.252	0.047
MCV, fL	65.7	70.3	70.4	69.7	65.3	0.6	0.833	<0.001
MCH, pg	20.4	21.0	21.1	21.3	20.7	1.0	0.431	0.163
TP, g/L	44.88	44.95	41.38	41.03	43.68	0.99	0.743	0.139
TC, mmol/L	2.48	2.61	2.21	2.26	2.47	0.06	0.193	0.152
TBIL, μmol/L	3.46	4.06	2.84	3.72	3.12	0.18	0.414	0.699

^1^ 0NAM: experimental diet without niacinamide; 30NAM: diet supplemented with 30 mg/kg niacinamide; 130NAM: diet supplemented with 130 mg/kg niacinamide; 230NAM: diet supplemented with 230 mg/kg niacinamide; 330NAM: diet supplemented with 330 mg/kg niacinamide. Quad, quadratic; WBC, white blood cell; LYM, lymphocytes; MON, monocytes; GRA, granulocytes; HGB, haemoglobin; MCV, mean erythrocyte volume; MCH, haemoglobin content. TP, total protein; TC, triglyceride; TBIL, total bilirubin. SEM: standard error of the means (*n* = 6). *p*-value: *p* < 0.05 indicating significant differences and 0.05 ≤ *p* < 0.10 meaning a trend.

**Table 6:** Serum physiological parameters, oxidative stress, and immune-related indicators of nursery pigs fed diets with increasing levels of niacinamide (Exp. 2) ^1^.

Items	Treatments				SEM	*p*-Value	
	30N	130N	230N	330N		Linear	Quad
WBC, ×10^9^/L	21.0	16.6	19.4	23.4	3.0	0.487	0.303
LYM, %	33.3	44.6	34.5	28.5	3.1	0.374	0.101
MON, %	7.6	9.6	7.8	8.1	1.3	0.995	0.822
GRA, %	59.1	45.8	57.8	63.5	3.8	0.456	0.131
HGB, g/L	88.8	100.5	94.5	93.3	3.1	0.762	0.049
MCV, fL	59.1	61.3	58.9	60.9	1.2	0.577	0.860
MCH, pg	17.1	17.1	16.8	17.4	0.3	0.649	0.331
ALB, g/L	31.0	32.3	31.3	32.4	1.1	0.392	0.694
TP, g/L	51.5	51.4	51.5	51.8	1.7	0.892	0.984
TC, mmol/L	2.4	2.4	2.2	2.2	0.2	0.121	0.299
TBIL, μmol/L	3.9	3.2	3.5	3.7	0.8	0.789	0.777
SOD, pg/ml	251	268	243	231	12	0.358	0.546
GSH-Px, pmol/ml	66	71	58	64	7	0.414	0.914
MDA, nmol/L	1.3	1.5	1.2	1.4	0.2	0.938	0.986
TNF-α, pg/ml	365	353	404	286	36	0.284	0.198
IL-6, ng/L	1061	1141	855	1026	98	0.387	0.576
IgG, μg/ml	472	499	435	449	42	0.457	0.767
IgA, μg/ml	39	44	47	37	2	0.450	0.006

^1^ 30N: diet containing 30 mg/kg niacinamide supplementation; 130N: diet containing 130 mg/kg niacinamide supplementation; 230N: diet containing 230 mg/kg niacinamide supplementation; 330N: diet containing 330 mg/kg niacinamide supplementation. Quad, quadratic; WBC, white blood cell; LYM, lymphocytes; MON, monocytes; GRA, granulocytes; HGB, haemoglobin; MCV, mean erythrocyte volume; MCH, haemoglobin content; ALB, albumin; TP, total protein; TC, triglyceride; TBIL, total bilirubin; SOD, superoxide dismutase; GSH-Px, glutathione peroxidase; MDA, malondialdehyde; TNF-α, tumor necrosis factor-α; IL-6, interleukin-6; IgG, immunoglobulin G; IgA, immunoglobulin A; SEM: standard error of the means (*n* = 4). *p*-value: *p* < 0.05 indicating significant differences and 0.05 ≤ *p* < 0.10 meaning a trend.

**Table 7:** The intestinal structure of nursery pigs fed diets with increasing levels of niacinamide (Exp. 2) ^1^.

Items	Treatments				SEM	*p*-Value	
	30N	130N	230N	330N		Linear	Quad
Duodenum							
VH, um	363	382	463	488	16	0.001	0.002
CD, um	267	255	290	277	8	0.290	0.792
V/C	1.36	1.50	1.60	1.76	0.07	0.029	0.341
Jejunum							
VH, um	330	357	373	383	14	0.037	0.094
CD, um	266	218	254	206	12	0.254	0.987
V/C	1.24	1.64	1.47	1.86	0.07	0.191	0.591
Ileum							
VH, um	342	385	386	375	16	0.548	0.446
CD, um	258	188	185	229	10	0.245	0.017
V/C	1.33	2.05	2.09	1.64	0.10	0.682	0.005

^1^ 30N: diet containing 30 mg/kg niacinamide supplementation; 130N: diet containing 130 mg/kg niacinamide supplementation; 230N: diet containing 230 mg/kg niacinamide supplementation; 330N: diet containing 330 mg/kg niacinamide supplementation. Quad, quadratic; VH, villus height; CD, crypt depth; V/C, villus height to crypt depth ratio; SEM: standard error of the means (*n* = 4). *p*-value: *p* < 0.05 indicating significant differences and 0.05 ≤ *p* < 0.10 meaning a trend.

**Table 8:** The colonic short-chain fatty acid of nursery pigs fed diets with increasing levels of niacinamide (Exp. 2) ^1^.

Items	Treatments				SEM	*p*-Value	
	30N	130N	230N	330N		Linear	Quad
TVFA, mmol/L	28.92	39.98	35.06	41.03	1.17	0.003	0.183
Acetic acid, mmol/L	18.20	22.60	22.22	25.77	0.77	0.001	0.753
Propionic acid, mmol/L	7.57	11.77	8.61	10.30	0.51	0.304	0.138
Butyric acid, mmol/L	2.41	3.94	3.35	3.70	0.17	0.048	0.063
Isobutyric acid, mmol/L	0.12	0.09	0.11	0.13	0.02	0.405	0.406
Valeric acid, mmol/L	0.51	0.83	0.67	0.80	0.09	0.203	0.278
Isovaleric acid, mmol/L	0.11	0.12	0.10	0.17	0.02	0.187	0.229
Acetic acid/propionic acid	2.38	2.02	2.70	2.54	0.18	0.149	0.337

^1^ 30N: diet containing 30 mg/kg niacinamide supplementation; 130N: diet containing 130 mg/kg niacinamide supplementation; 230N: diet containing 230 mg/kg niacinamide supplementation; 330N: diet containing 330 mg/kg niacinamide supplementation. Quad, quadratic; TVFA, total volatile fatty acids; SEM: standard error of the means (*n* = 4). *p*-value: *p* < 0.05 indicating significant differences and 0.05 ≤ *p* < 0.10 meaning a trend.

## Data Availability

The data presented in this study are available from the corresponding author upon reasonable request.
